# Identification of asthma control factor in clinical notes using a hybrid deep learning model

**DOI:** 10.1186/s12911-021-01633-4

**Published:** 2021-11-09

**Authors:** Bhavani Singh Agnikula Kshatriya, Elham Sagheb, Chung-Il Wi, Jungwon Yoon, Hee Yun Seol, Young Juhn, Sunghwan Sohn

**Affiliations:** 1 Department of Artificial Intelligence and Informatics, Mayo Clinic, 200 First St SW, Rochester, MN 55905 USA; 2 Precision Population Science Lab, Department of Pediatric and Adolescent Medicine, Mayo Clinic, Rochester, MN USA; 3 Department of Pediatrics, Myongji Hospital, Goyang, South Korea; 4 Pusan National University, Yangsan Hospital, Yangsan, South Korea

**Keywords:** Deep learning, Context-aware language model, Natural language processing, Documentation variations, Adherence to asthma guidelines, Inhaler technique

## Abstract

**Background:**

There are significant variabilities in guideline-concordant documentation in asthma care. However, assessing clinician’s documentation is not feasible using only structured data but requires labor-intensive chart review of electronic health records (EHRs). A certain guideline element in asthma control factors, such as review inhaler techniques, requires context understanding to correctly capture from EHR free text.

**Methods:**

The study data consist of two sets: (1) manual chart reviewed data—1039 clinical notes of 300 patients with asthma diagnosis, and (2) weakly labeled data (distant supervision)—27,363 clinical notes from 800 patients with asthma diagnosis. A context-aware language model, Bidirectional Encoder Representations from Transformers (BERT) was developed to identify inhaler techniques in EHR free text. Both original BERT and clinical BioBERT (cBERT) were applied with a cost-sensitivity to deal with imbalanced data. The distant supervision using weak labels by rules was also incorporated to augment the training set and alleviate a costly manual labeling process in the development of a deep learning algorithm. A hybrid approach using post-hoc rules was also explored to fix BERT model errors. The performance of BERT with/without distant supervision, hybrid, and rule-based models were compared in precision, recall, F-score, and accuracy.

**Results:**

The BERT models on the original data performed similar to a rule-based model in F1-score (0.837, 0.845, and 0.838 for rules, BERT, and cBERT, respectively). The BERT models with distant supervision produced higher performance (0.853 and 0.880 for BERT and cBERT, respectively) than without distant supervision and a rule-based model. The hybrid models performed best in F1-score of 0.877 and 0.904 over the distant supervision on BERT and cBERT.

**Conclusions:**

The proposed BERT models with distant supervision demonstrated its capability to identify inhaler techniques in EHR free text, and outperformed both the rule-based model and BERT models trained on the original data. With a distant supervision approach, we may alleviate costly manual chart review to generate the large training data required in most deep learning-based models. A hybrid model was able to fix BERT model errors and further improve the performance.

## Background

Asthma is the most common chronic illness among children as well as one of the five most burdensome adult chronic diseases in United States (US), causing significant morbidity and cost [1–3]. Implementation of and adherence to the asthma guidelines have been reported to improve asthma care and outcomes [4–8]. However, there are significant variabilities in clinicians’ guideline adherence (i.e., documentation of patients’ asthma-related conditions recommended in asthma guidelines) in asthma care [9–11]. The clinical documentation in free text is an essential component in electronic health records (EHRs) and the quality of information resided in clinical documents plays a critical role in patient care, clinical research, and quality assurance in health care. Clinicians are the primary creator of EHRs, but their documentation varies substantially [12], which may significantly affect the quality of both structured and unstructured data and cause a potential bias for their downstream applications. Therefore, improving the quality of clinical documentation by assessing clinician’s adherence to guidelines to care management (e.g., asthma management guidelines) in documentation is crucial not only to maximize the meaningful use of EHRs but also to improve clinical practice.

The 2007 National Asthma Education and Prevention Program (NAEPP) asthma guidelines provides guidance for improved asthma management using asthma control, factors with control, and medications [11]. Although more than 10 years had been passed since NAEPP asthma guidelines, clinician’s low adherence to asthma guidelines have been widely recognized. However, assessing clinicians’ adherence to asthma guidelines is not feasible using only structured data but requires manual chart review of EHRs, which is labor-intensive, time consuming, and costly [11, 13]. Since certain guideline elements of NAEPP are not available through structured data, the advanced techniques, such as natural language processing (NLP), is required to mitigate the issues of time-consuming manual chart review. Some guideline elements in EHR free text are relatively straightforward (e.g., asthma medications, daytime and nighttime symptoms) and can be identified by handcrafted rules based on keywords and description patterns. However, a certain guideline element regarding asthma control status, such as teaching and reviewing inhaler techniques, requires context (semantic) understanding to capture from EHR free text, not suitable to be handled by rules. For example, inhaler techniques (i.e., teaching patients how to use an asthma inhaler or reviewing their inhaler use), “A patient received asthma education and instruction in appropriate metered-dose inhaler technique.”; “Discussed 3rd neb treatment here versus one upon home.” The first example is semantically correct for teaching inhaler techniques but the second one is not necessarily implying teaching and reviewing inhaler techniques but discussing efficacy of neb treatment, which has to be differentiated from the intended concept (reviewing and teaching true inhaler techniques).

Recent advances in deep learning using contextual embedding have a capability to better understand context (i.e., semantic meaning of words or phrases), which may allow capturing complex concepts such as clinician’s adherence to asthma guideline elements which could be often missed by handcrafted rules. Especially, a context-aware language model, Bidirectional Encoder Representations from Transformers (BERT) [14] is a pre-trained NLP framework that has promising results on a variety of NLP tasks. However, in general, a deep learning model requires a large number of labeled data to learn the tasks properly in order to perform well. Manual chart review is considered as a gold standard to generate labeled data but it requires significant amount of labor, time, and cost. Often this is a critical bottleneck in development of a deep learning-based model in the clinical domain. There is great need to address this challenge. A distant supervision, which produces labeled data using rules or heuristics to train the model, has been successfully used to alleviate this challenge [15, 16]. Rules or heuristics reflecting expert knowledge may automatically generate the reasonably labeled data to augment the training data in a way a deep learning model better learns the underlying concepts to be captured from a distantly supervised large data set.

This study applied BERT-based models to examine the feasibility of identifying an asthma control factor, inhaler techniques from EHR free text (clinical notes) and further investigated the effect of BERT-based models incorporated with distant supervision. A hybrid approach using post-hoc rules was also tested to fix BERT-based model errors and improve the performance. The performance of BERT-based and hybrid models was also compared with a rule-based approach.

## Literature review (BERT in clinical applications)

Rule-based NLP techniques have been successfully applied in asthma research with high performance [17–21]. A rule-based approach has been used widely in the clinical domain to implement existing criteria with expert knowledge. It is relatively flexible to customize and tolerant to imbalance data. However, a rule-based approach needs significant effort until it reaches to high performance [22, 23].

Recently, the deep learning-based models have become more frequent than traditional machine learning models in the clinical domain due to availability of large data, diversified algorithms, and the technical abilities to handle complex algorithms [24]. However, there are some limitations using these models dealing with complex long-term dependencies, shallow networks, training from scratch and others. Recent advancements in NLP developed new learning representation models that consider not only complex contextual representations but also the ability to use pretrained models [25]. Out of these, BERT has outperformed other models and produced state-of-the art results in many NLP tasks due to its large pretrained contextual information [14, 26]. BERT has gained popularity in its application in both clinical and biomedical domain due to its superior performance and ability to use pretrained word representations in downstream tasks. In clinical domain, BERT has demonstrated high performance in clinical relation extraction tasks, classification of clinical texts, and phenotyping of clinical notes [27–30]. Researchers have developed pretrained embeddings related to various domains including clinical BERT, which was trained on Medical Information Mart for Intensive Care III (MIMIC-III) notes [31–33], and clinical BioBERT (cBERT), which was trained on MIMIC-III clinical notes but was initialized on BioBERT and outperformed other BERT models in majority of clinical tasks suggesting the advantage of cBERT in clinical applications [33].

This study used both original BERT and cBERT with and without distant supervision to identify inhaler techniques that require context understanding and to examine if a domain specific pretrained model (cBERT) outperforms general pretrained embeddings model (BERT) in our classification tasks. We also evaluate a hybrid approach to examine if a BERT-based model on verge of overfitting or undertraining can be improved by adding simple post-hoc rules on its prediction.

## Methods

Our task is to identify the description of reviewing/teaching inhaler technique (Table 1) in clinical notes. Since the aim of the study is to assess clinician’s adherence to asthma guidelines (e.g., whether clinicians documented inhaler technique reviewed or taught in clinical notes), any documentation regardless of negation was considered as guideline congruent.

**Table 1:** Examples of inhaler techniques in NAEPP

Required documentation	Example
Teaching or review	Discussed correct inhaler technique
	A patient received asthma education and instruction in appropriate metered-dose inhaler technique

### Data

The study data consists of two data sets: (1) manual chart reviewed data—1039 clinical notes of 300 patients with asthma diagnosis, randomly selected from the Olmsted County birth cohort (2016–2018). We used notes from 200 patients as a training set (n = 724) and notes from the other 100 patients (n = 315) as a test set. A physician performed chart review and annotated guideline elements based on 2007 NAEPP guidelines; and (2) weakly labeled data (distant supervision)—27,363 clinical notes from 800 patients with asthma diagnosis that were randomly selected from the Olmsted County birth cohort (2008–2018) for the training set. The guideline elements were labeled by handcrafted rules (Table 2) instead of manual chart review (i.e., weakly labeled). This data is to train BERT and cBERT models with distant supervision. We used the same test set as in the manual chart reviewed data to compare the performance of inhaler technique identification. We used the contents in the note sections of History of Present Illness and Impression/Report/Plan sections of EHRs since majority of teaching and reviewing inhaler techniques reside in these sections.

**Table 2:** Rules to identify inhaler techniques

Keywords	Rules
(1) Observ*, reassess*, review*, demonstrat*, check*, educat*, teach*, taught, explain*, reinforce*, discuss*, instruction, constraints, how to use	(a) Combination of keyword 1 and 2 (e.g., reviewed inhaler use)
(2) Inhaler, MDI, neb, nebulizer, optichamber, spacer	
(3) Techniques, administrations, dosing, guidance	(b) Combination of keyword 1, 3, and 4 (e.g., teach daily medication technique)
(4) Asthma/rescue/daily/preventive/control medication, ICS, list of maintenance and rescue medications	

* (Asterisk):  match any characters

### Rule-based model

The rules were developed by using common patterns based on textual markers (i.e., keywords relevant to asthma guideline elements) and evaluated against manual chart review as a gold standard. The keywords were provided by domain experts and updated and refined iteratively as we developed rules on the training set (Table 2). We implemented rules under the framework of MedTaggerIE [34], a clinical NLP pipeline developed by Mayo Clinic. The performance of the NLP algorithm was evaluated in a document level (i.e., whether a guideline element is recorded or not in the clinical note) since the guideline adherence in asthma care is measured in a document level.

### BERT and cBERT

A ‘bert-base-uncased’ was used in a BERT due to its good performance with less computational requirements. The model was built with the addition of a dropout layer (*p* = 0.1) and a linear classification layer with cross entropy loss function to perform binary classification (i.e., presence or absence of inhaler technique). The BERT tokenizer was used to tokenize clinical notes and padded all the input sentences (maximum sequence length = 256) as all the sentences in our data had tokens less than 256. Each sentence is labelled as presence or absence of inhaler technique, and then a document-level classification was performed by examining sentences within a given document—i.e., presence of inhaler technique if exists any sentences with inhaler technique; absence of inhaler technique if there is no sentence with inhaler technique in a given document. We implemented a cyclical learning rate with triangular mode scheduler ranging between lower bound of 2e−5 and upper bound of 5e−5 with a step size of 2500 and initialized at 3e−5 learning rate. The model was trained, validated (10 epochs), and tested on manual chart reviewed data. A part of the training set (about 12%) was used as validation set for inhaler technique. In the training set (sentences), only 0.4% of them is presence of inhaler techniques and the test has 0.56% presence of inhaler techniques. The data sets are highly imbalanced and thus the cost sensitivity approach [35] was used to deal with the imbalanced data issue; we set the weights in our cross entropy loss function and incorporated it into our BERT model to penalize more towards misclassification on minority samples.

The cBERT differs in the pretrained contextual representations from the BERT and has vocabulary size of 28,996. This model was implemented using similar cyclical learning rate and cost sensitivity approaches similar to BERT.

### BERT and cBERT with distant supervision (BERT-DS and cBERT-DS)

**Fig. 1:**
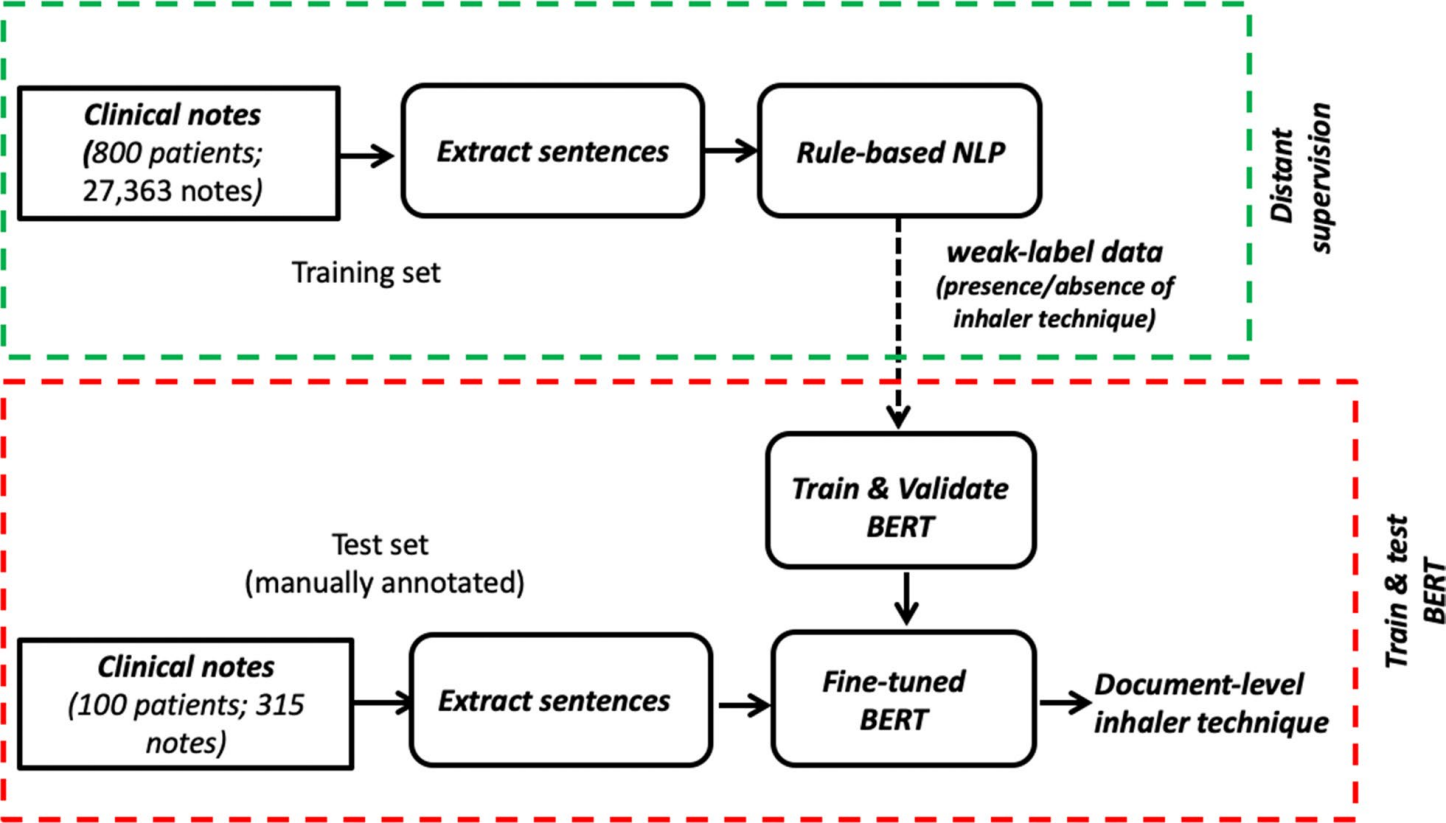
BERT-based models with distant supervision

Figure 1 shows an overview of the process. The weakly labeled data, which used a rule-based model (“Rule-based model” section) to label presence or absence of inhaler technique, were used to train the BERT and cBERT model. These weak-labeled trained models are referred to as BERT-DS (BERT trained on distant supervised data [i.e., weak-labeled data]) and cBERT-DS (cBERT trained on distant supervised data). The training sets (sentences) are significantly imbalanced with 0.124% of presence of inhaler techniques, necessitating the use of cost sensitivity to penalize more on the minority misclassification. The cost sensitive weights were determined experimentally to [0.52, 5.52]. The model was trained for 10 epochs with the learning rate of 3e−5 and tested on the same manual chart reviewed data (“BERT and cBERT” section).

### Hybrid model

A hybrid model used post-hoc rules to fix trivial errors from BERT-DS and cBERT-DS outcomes—i.e., applied simple rules on the results from BERT-DS and cBERT-DS to overwrite class labels. The post-hoc rules were adapted from the original rules (a simplified version with relaxed or strict conditions) based on error analyses on the training set of a BERT model (i.e., by examining discrepancies between gold standard labels and BERT model outcomes). The rationale of using post-hoc rules is that a BERT model might incorrectly behave on relatively simple problems due to over- or under-fitting to the data, and this error might be corrected by rules.

Table 3 contains post-hoc rules we implemented. The rules denoted by N2P were applied on negative outcomes of BERT-DS and cBERT-DS (i.e., absence of inhaler technique) and overwrote the label to positive (i.e., presence of inhaler technique) if rules were satisfied; the rules denoted by P2N were applied on the positive outcomes of BERT-DS and cBERT-DS and overwrote the label to negative if rules were satisfied.

**Table 3:** Post-hoc rules in a hybrid model

Inhaler technique
**N2P:**
(instructed|reviewed|discussed).*(use\sof\sMDI|use\sof\sspacer|use\sof\snebulizer|use\sof\sinhaler)
**P2N:**
(discussed|discussion).* AND no indication of “technique”

*N2P* negative to positive, *P2N* positive to negative

. (Dot): match any characters, * (Asterisk): match zero or more occurrences

## Results

The performance of inhaler technique identification was compared among different models: rule-based, BERT/cBERT (trained on the small manual chart reviewed data), and BERT-DS/cBERT-DS (distant supervision; trained on the large weakly labeled data), and hybrid models (BERT-DSH and cBERT-DSH; post-hoc rules applied on BERT-DS and cBERT-DS respectively). We calculated precision, recall, various F-score (F1, F2, and F0.5), and accuracy on the test set as evaluation metrics. F1-score is a balanced F-score using harmonic mean of precision and recall; F2-score weighs recall higher than precision (i.e., false negative is more costly); F0.5-score weighs precision higher than recall (i.e., false positive is more costly).

Table 4 shows the performance among different models. The BERT and cBERT trained on the small data had similar (slightly higher) overall performance (F1-score and accuracy) compared to the rule-based model. When the distant supervision was applied (i.e., trained on the larger weakly labeled data), it produced higher performance in F1-score and accuracy than those of the rule-based and the original BERT and cBERT models trained on the small data. Although cBERT did not perform better than BERT, cBERT-DS had higher F1-score and accuracy than BERT-DS. The use of post-hoc rules on BERT-DS and cBERT-DS (a hybrid model; BERT-DSH and cBERT-DSH) further improved the performance. F2- and F0.5-score were also used to compare the different weights of false positives and false negatives. cBERT had higher F2-score but lower F0.5-score than BERT. All models costed more on false positives (i.e., F0.5-score > F2-score). Overall, cBERT-DSH performed highest for all three F-scores.

**Table 4:** Performance of inhaler technique identification

Model	Precision	Recall	F1-score	F2-score	F0.5-score	Accuracy
Rules	0.861	0.815	0.837	0.824	0.851	0.961
BERT	0.909	0.789	0.845	0.810	0.882	0.965
cBERT	0.861	0.816	0.838	0.825	0.852	0.961
BERT-DS	0.865	0.842	0.853	0.847	0.860	0.965
cBERT-DS	0.892	**0.868**	0.880	0.873	0.887	0.971
BERT-DSH	0.914	0.842	0.877	0.855	0.899	0.971
cBERT-DSH	**0.942**	**0.868**	**0.904**	**0.882**	**0.926**	**0.978**

Bold: best performance for a given metric

Actual cases of inhaler technique identification among different models were compared and analyzed (Table 5). The models using BERT and cBERT were able to correctly identify cases that are false positive (FP) by the rule-based model (Example A). These cases require to understand semantic of free text, which is difficult to handle by handcrafted rules. The rule-based model used handcrafted rules developed on the training set—i.e., keywords and their combinations in certain patterns. Explicit expressions of inhaler techniques can be handled by these rules. However, inhaler techniques are often described in indirect or complex forms of expression that require context understanding to correctly determine true inhaler techniques, not simply by presence/absence of keywords. The handcrafted rules can capture any expressions that contain relevant keyword combinations but were not able to understand context to accurately discern false positive cases, which contains keywords but not true inhaler technique semantically, leading to relatively low precision. For example, “We reviewed her medications and discussed labeling the Flovent with a green sticker for a daily inhaler…” This sentence contains keywords ‘discussed’ and ‘inhaler’ thus determined as inhaler techniques by rules. However, this does not refer to reviewing or teaching inhaler technique but discussing approaches improving compliance (i.e., false positive). Likewise, a rule-based model missed the cases that does not include relevant keywords (Example C); there is no explicit keyword related to review or teaching thus missed by rules but captured by the models using BERT-based models.

**Table 5:** Comparison of inhaler technique identification among different models

Examples	Rules	BERT	cBERT	BERT-DS/Hybrid	cBERT-DS/Hybrid
*A. Absence of inhaler technique*					
Reviewed importance of twice daily dosing of Flovent	FP	C	C	FP/FP	C/C
We reviewed her medications and discussed labeling the Flovent with a green sticker for a daily inhaler	FP	C	C	FP/C	FP/C
*B. Presence of inhaler technique*					
Reinforced the importance of using the chamber and mask to deliver	C	FN	FN	C	C
Mom voices no concerns about his technique in using the inhaler	C	FN	FN	C	C
*C. Presence of inhaler technique*					
Reinforced spacer use	FN	C	C	C	C
*D. Presence of inhaler technique*					
XXXX was also aided with an appropriate sized mask, and instructed	FN	FN	FN	FN/C	FN/C
*E. Presence of inhaler technique*					
I also did some training today regarding the use of albuterol with a spacer	FN	C	FN	FN	FN
Michelle is using a mask and a spacer appropriately	FN	C	FN	FN	FN
*F. Presence of inhaler technique*					
She is comfortable spacer technique	FN	FN	C	FN	FN
Our nurse, went in before me and checked on his technique and compliance	FN	FN	C	FN	FN
*G. Presence of inhaler technique*					
Was given an appropriate fitting mask, and discussed Pulmicort nebs, as XXXX was resistant to her MDI	FN	FN	FN	FN	FN

*FP* false positive, *FN* false negative, *C* correct

BERT-DS and cBERT-DS were able to identify cases missed by BERT and cBERT (Example B) but there are opposite cases as well (Example E, F). It was evident that a hybrid model could correct errors in multiple examples (Example A, D). Also, there are cases that all models failed to correctly identify inhaler techniques (Example G). The more diverse training data seem to be required for BERT-based models to identify these challenging cases.

## Discussion

Reviewing and teaching asthma inhaler technique is an important factor of asthma control in NAEPP but reported low clinicians’ adherence. The assessment of clinician’s adherence to reviewing inhaler technique requires manual chart review or necessitates advanced techniques to understand semantics in clinical free text in order to automate the process. This study addresses this need by developing a deep learning-based model (BERT-based models) coupled with distant supervision and post-hoc rules (hybrid approach).

A rule-based model is transparent, easily customizable, and suitable to capture relatively simple and clear concepts with explicit keywords, but labor-intensive requiring expert knowledge. It is less likely suffered from imbalance data but difficult to capture semantics of true inhaler techniques, often causing false positive cases (relatively lower precision than BERT-based models). This is because rules are based on presence of keywords (e.g., discuss, nebulizer) but lack contextual understanding to discern unrelated cases as seen in Table 5. A BERT-based model demonstrated a capability to overcome the limitation of a rule-based model but it requires large enough data to outperform the rules. As can be seen in Table 4, BERT-based models (BERT, cBERT) trained on the small data set (same data as the rule-based model) showed similar performance compared to the rule-based model but they can avoid labor-intensive rule development. A distant supervision is a promising way to generate large data without costly manual annotation for the training set. BERT-based model using weakly labeled data outperformed both a rule-based and original BERT-based models on the small data, and may be further improved when applied on larger data.

Interestingly, the patterns or semantics learned by BERT-based models on the small data by manual chart review seem to be different from BERT-based models with distant supervision, which is evident from several examples. This may be because the weakly label data generated by rules were biased on explicit existence of reviewing/teaching indications and inhaler keywords (e.g., “reviewed inhaler use”), whereas the small data (manual chart reviewed data) contain relatively more portion of implicit descriptions in EHRs. All models failed to identify certain cases that do not contain explicit indications but require rather semantic inference (Table 5 Example G). More data with diverse semantic expressions would be needed for BERT-based models to better learn these problems. A hybrid approach using post-hoc rules on top of BERT-based models further improved the performance. This indicates that post-hoc rules could be used as a complimentary to fix some BERT-based model errors, diminishing the issue of the model generalizability or specialization due to limited data.

The BERT-based model (including a hybrid model) can be used to automatically assess clinicians’ adherence to asthma guidelines (i.e., clinicians’ documentation regarding asthma-related information of patients) which often reflects care quality for asthma. Under the current pay for performance or value-based care policy promoting health care organizations to achieve the quadruple aim for value-based care (improve care quality, user experience and health outcomes; reduce health care costs), availability of the efficient and effective (i.e., automated) method of measuring care quality such as clinician’s adherence to guidelines beyond structured data (e.g., ED visit for asthma) can be instrumental. In this respect, the presented work lays an important foundation for optimizing asthma care and research in the future.

In addition, the model can also discover the variability of adherence of individual clinicians to derive evidence from their actual clinical practice reflected in EHR documents. This variation in documenting asthma-related events in EHRs impacted health outcomes (e.g., increased ED visits) and also has an important clinical implication such as a proficiency in achieving clinical competence in asthma care and documentation. Thus, this individualized digital assistant has a potential to improve accuracy and quality of clinical documentation as well as better guide clinicians’ monitoring asthma care. The medical education requires the provision of data-driven clinical practice derived from actual clinical notes. However, many programs lack tools to this assessment because manual chart review of clinical notes is challenging. Our models can help address this challenge and provide personalized clinical effectiveness data derived from clinical documentation, improving clinician’s adherence to asthma guidelines and ultimately better optimize asthma care and education.

There are several limitations in this study. We observed some inconsistency in manual annotation of inhaler technique and reconciled them during the model development. However, there still might be some inconsistent annotation in the data due to the intrinsic complexity of the definition of clinician’s adherence behavior and documentation for inhaler techniques in EHRs. Although this inconsistency often exists in clinical NLP tasks due to ambiguity in problem definition, it may affect the model’s behavior. Some incorrect weak labels generated by rules might hinder efficacy of distant supervision, but rules performed reasonably well to augment the training set and further improve the performance of BERT-based models. The hybrid approach that overrides the prediction of BERT-based models might correct some of these issues. Our models were developed using clinical notes in a single institution tailored to a specific EHR system. Although we achieved high performance, the models may not perform similarly on EHR data from institutions due to EHR variabilities.

## Conclusions

A deep learning approach (BERT-based models) with distant supervision (i.e., trained on weakly labeled data) demonstrated the capability to identify inhaler techniques, which require semantic understanding in clinical narratives, and outperformed both the rule-based model and the original BERT-based models on the small data. With a distant supervision approach, we may alleviate costly manual chart review to generate the training data required in most deep learning-based model. The use of post-hoc rules in a hybrid approach further improved the performance of BERT-based models, indicating a potential as a complimentary in deep learning model development when there is not enough training data to correctly learn all patterns. The proposed approach might be a potential alternative to a rule-based model.

## Data Availability

The data are not publicly shareable as they contain protected health information.

## Abbreviations

BERT: Bidirectional encoder representations from transformers
cBERT: Clinical BioBERT
EHRs: Electronic health records
NAEPP: National asthma education and prevention program
NLP: Natural language processing
